# Not all questions are created equal: the weight of the Oxford Knee Scores questions in a multicentric validation study

**DOI:** 10.1186/s10195-023-00722-6

**Published:** 2023-08-17

**Authors:** Matthias Luger, Clemens Schopper, Eliana S. Krottenthaler, Mahmoud Mahmoud, Thomas Heyse, Tobias Gotterbarm, Antonio Klasan

**Affiliations:** 1 Department for Orthopedics and Traumatology, Kepler University Hospital GmbH, Krankenhausstrasse 9, 4020 Linz, Austria; 2 Johannes Kepler University Linz, Altenberger Strasse 69, 4040 Linz, Austria; 3 Red Cross Hospital Frankfurt Germany, Königswarterstraße 16, 60316 Frankfurt am Main, Germany; 4 AUVA UKH Steiermark, Göstinger Strasse 14, 8020 Graz, Austria; 5 Kepler University Hospital Linz, Krankenhausstrasse 9, 4020 Linz, Austria

**Keywords:** Oxford Knee Score, OKS, Knee arthroplasty, TKA, PROMs, Knee surgery

## Abstract

**Background:**

The Oxford Knee Score (OKS) has been designed for patients with knee osteoarthritis and has a widespread use. It has 12 questions, with each question having the same weight for the overall score. Some authors have observed a significant ceiling effect, especially when distinguishing slight postoperative differences. We hypothesized that each questions’ weight will depend significantly on the patient’s sociodemographic data and lifestyle.

**Methods:**

In this international multicentric prospective study, we included patients attending a specialist outpatient knee clinic. Each patient filled out 3 questionnaires: (a) demographic data and data pertaining to the OKS, (b) the standard OKS, and (c) the patient gave a mark on the weight of the importance of each question, using a 5-point Likert scale (G OKS). Linear regression models were used for the analysis.

**Results:**

In total 203 patients (106 female and 97 male) with a mean age of 64.5 (±12.7) years and a mean body mass index (BMI) of 29.34 (±5.45) kg/m^2^ were included. The most important questions for the patients were the questions for pain, washing, night pain, stability, and walking stairs with a median of 5. In the regression models, age, gender, and driving ability were the most important factors for the weight of each of the question.

**Conclusion:**

The questions in the OKS differ significantly in weight for each patient, based on sociodemographic data, such as age, self-use of a car, and employment. With these differences, the Oxford Knee Score might be limited as an outcome measure. Adjustment of the OKS that incorporates the demographic differences into the final score might be useful if the ceiling effect is to be mitigated.

*Level of Evidence:* Level II prospective prognostic study

## Introduction

The Oxford Knee Score (OKS) was first described by Dawson et al. [1] in 1997 and is one of the most common patient-reported outcome measures (PROM). The OKS was specifically developed and validated for measuring outcomes of knee replacement surgery, designed to be used pre- and postoperatively [1, 2]. The OKS [1] consists of 12 questions assessed on a Likert scale, each question valued from 0 to 4. A summative score is calculated, with 48 points as the best possible score (least symptomatic) and 0 points as the worst possible score (most symptomatic) [1–3].

Patients who underwent a knee arthroplasty are in general satisfied in terms of pain reduction and overall improved function, due to improvements in materials, surgical technique, and fixation [6–8]. But in the past 20 years, the dissatisfaction has remained stagnant at 10–20% [9]. Furthermore, the improvements resulted in ceiling effects in commonly applied PROMs, including OKS and WOMAC [10, 11]. Ceiling or floor effects occur when a considerable number of patients score the maximum or minimum score [2]. As a result, the outcome measure is unable to discriminate between subjects at the top or bottom of the scale [12, 13].

PROMs are also often unable to detect subtle differences in patient satisfaction between different designs or implantation techniques [11]. Alternative alignment strategies such as kinematic alignment are increasingly used to improve patient outcome. Results are mixed without clear statistically significant differences in postoperative outcome between different alignment techniques [14]. To detect subtle differences, new PROMs were subsequently developed, such as the Forgotten Joint Score 12 (FJS-12) [11]. The FJS-12 is only used postoperatively and aims at different questions compared with established scores such as the OKS or Western Ontario and McMaster Universities Osteoarthritis (WOMAC) score [11], with significantly lower ceiling effects compared with the OKS [15].

The OKS rates each of its question with the same numeric value. We hypothesized that each question does not have the same weight for each patient and that the weight depends significantly on the patient’s sociodemographic data and lifestyle. Therefore, this study was conducted to evaluate the weight of all questions of the OKS in patients with osteoarthritis of the knee.

## Material and methods

### Study setting and design

This prospective multicentric cohort study recruited patients coming to two specialist outpatient knee clinics in two German-speaking countries. The patients attended the clinics because of symptoms associated to osteoarthritis of the knee. All patients received X-rays of the knee to validate the osteoarthritis of the knee. The patients were asked to complete three questionnaires, in this consecutive order: First, they provided demographic data (age, gender, height, weight, and BMI) as well as data pertaining to the OKS. At this point, the patient was blinded to the subsequent questionnaires (stairs at home, self-use of a car, use of walking aids, working status, and previous surgery). Then, the patient filled out the standard validated OKS in the German language [1, 4]. Finally, the patient gave a grade to the personal importance of each question’s topic, using a 5-point Likert scale (G OKS).

### Ethics

Ethics approval was obtained from the local ethics committee in both centers (1286/2020 and 2021-2439-evBO) prior to conducting the study. All patients gave informed consent for participating in this study.

### Statistical analysis

Data analysis was performed using SPSS version 27.0 (IBM SPSS statistics, Armonk, NY, USA). A power analysis was performed to achieve a 0.3 Pearson correlation coefficient using a beta of 0.8 and an alpha of 0.05. At least 85 patients were required. Descriptive analysis was conducted for patient demographics. Normality distribution was assessed using the Shapiro–Wilk test. Normally distributed data is presented using mean [± standard deviation (SD)], whereas non-normally distributed data are presented using median [interquartile range (IQR)]. Testing for differences in patient demographics between both centers was done with Mann–Whitney-*U*-test for continuous variables and with Fisher’s exact test for categorical variables. A 5-point Likert scale was used for measuring the importance of each question (Q) of the OKS. Linear regression models were created to predict the weight of each of the questions. A *p*-value of < 0.05 was defined as significant.

## Results

### Demographics

All patients screened for inclusion were included in the study. In total, 203 patients were included in this study, with a median age of 66 years [13] – 100 patients in Center A and 103 patients in Center B. The study group consisted of 106 female patients and 97 male patients with a median BMI of 28.10 [6.53] kg/m^2^; see Table 1. As for the OKS-related demographic data, 92.1% of patients had stairs at home, 71.4% were still driving a car, 26.6% were using walking aids, 33.5% were still working, and 62.6% underwent previous knee surgery (Table 1).

**Table 1:** Patient demographics for the general cohort separated by center; *p*-value for testing for difference between both centers

Patient demographics	Total	Center 1	Center 2	*p*-Value
Number of patients	203 (100.0%)	100 (49.3%)	103 (50.7%)	
Male	97 (47.8%)	49 (49%)	48 (46.6%)	0.732
Female	106 (52.2%)	51 (51%)	55 (53.4%)	
Age (median, in years)	66 ± 16	64.5 ± 17	68 ± 15	**< 0.001**
BMI (kg/m^2^)	28.10 ± 6.53	28.97 ± 7.56	28.1 ± 6	0.869
Height (cm)	168 ± 15	170 ± 16	168 ± 13	0.094
Weight (kg)	82 ± 23	82.5 ± 27.8	82 ± 16	0.574
Stairs	187 (92.1%)	89 (89%)	188 (92.6%)	0.053
Drivers	145 (71.4%)	71 (71%)	74 (71.8%)	0.894
Walking aids	54 (26.6%)	20 (20%)	34 (33%)	**0.036**
Working	68 (33.5%)	36 (36%)	33 (32%)	0.551
Previous surgery	127 (62.6%)	70 (70%)	55.7 (55.3%)	**0.031**

Bold values indicate signifiacant *p*-values

For demographic data, there were differences between both centers in age (*p* < 0.001), the use of walking aids (*p* = 0.036), and previous surgeries (*p* = 0.031; Table 1).

### Oxford Knee Score and scored weight

The highest average score in the OKS was found for pain, with 3.61 points, and for kneeling, with 3.29 points (Table 2). The lowest average scores were found for washing, with 1.56 points, and walking for a longer distance, with 1.90 points (Table 2). The most important questions for the patients were the questions for pain, washing, night pain, stability, and walking stairs, with a median of 5. The scores for the OKS and the Likert scale on importance are given in Table 2.

**Table 2:** Mean and median values for the questions of the OKS and their importance according to the 5-point Likert scale

	Category	Mean OKS	Median OKS	Mean G OKS	Median G OKS
OKS Q1	Pain	3.61	4	4.48	5
OKS Q2	Washing	1.56	1	4.54	5
OKS Q3	Transport	2.11	2	4.12	4
OKS Q4	Walking for a longer distance	1.90	2	4.12	4
OKS Q5	Standing up after eating	2.24	2	4.22	4
OKS Q6	Limping	2.53	3	4.20	4
OKS Q7	Kneeling	3.29	4	4.02	4
OKS Q8	Night pain	2.71	3	4.62	5
OKS Q9	Working	2.68	3	4.43	4
OKS Q10	Stability	1.96	2	4.49	5
OKS Q11	Shopping	1.96	2	4.28	4
OKS Q12	Walking stairs	2.41	3	4.43	5

### Predictive analysis

#### Question 1: Pain

In the regression models for the weight of each of the questions, pain was associated with older age (*p* = 0.009; *B* = −0.013) and gender (*p* = 0.015, *B* = 0.301; Table 4).

#### Question 2: Washing

Washing was associated with older age (*p* = 0.013, *B* = −0.010) and gender (*p* < 0.001, *B* = 0.387) in the regression model (Table 4).

#### Question 3: Transport

The question on transport was associated with the patients’ self-use of a car (*p* < 0.001; *B* = 0.707) in the regression model (Table 4).

#### Question 4: Walking for a longer distance

The question on walking was associated with older age (*p* = 0.041, *B* = −0.013), gender (*p* = 0.046, *B* = 0.347), height (*p* = 0.015, *B* = 0.022), and self-use of a car (*p* = 0.002, *B* = 0.496) in the regression model (Table 4).

#### Question 5: Standing up after eating

Getting up after eating was associated with older age (*p* = 0.009, *B* = −0.013) and self-use of a car (*p* = 0.031, *B* = 0.285) in the regression model (Table 4).

#### Question 6: Limping

Limping was associated with older age (*p* = 0.011, *B* = −0.016), gender (*p* = 0.031, *B* = 0.385), and self-use of a car (*p* = 0.037, *B* = 0.334) in the regression model (Table 4).

#### Question 7: Kneeling

In the regression model, kneeling was associated with the self-use of a car (*p* = 0.027, *B* = 0.394; Table 4). A significant negative correlation between the recorded OKS score and recorded OKS weight was found for the question on kneeling (*r* = −0.158, *p* = 0.024; Table 3).

**Table 3:** Predictive analysis between the recorded OKS score and recorded OKS weight (significant values in bold letters)

OKS	Q1	Q2	Q3	Q4	Q5	Q6	Q7	Q8	Q9	Q10	Q11	Q12
*p*-Value	0.079	0.182	0.120	0.108	0.468	0.703	**0.024**	**0.043**	**0.014**	**0.018**	0.071	0.691
*r*	0.124	−0.094	−0.110	−0.114	0.051	0.027	**−0.158**	**0.143**	**0.174**	**0.166**	−0.127	−0.028

#### Question 8: Night pain

A significant positive correlation between the recorded OKS score and recorded OKS weight was found for the question on night pain (*r* = 0.143, *p* = 0.043) (Table 3). In the regression model, night pain was associated with older age (*p* = 0.037, *B* = −0.009) (Table 4).

**Table 4:** Regression coefficient B and *p*-values for the regression models for each question of the OKS and age, gender, height, weight, BMI, stairs, driving, use of walking aids, working status, and previous knee surgeries

OKS		Age	Gender	Height	BMI	Stairs	Driving	Walking aids	Working	Previous surgery
Q1	*B*	**−0.013**	**0.301**	0.002	−0.003	0.014	0.126	0.145	0.146	−0.059
	*p*-Value	**0.009**	**0.015**	0.796	0.757	0.945	0.346	0.263	0.291	0.601
Q2	*B*	**−0.010**	**0.387**	0.009	−0.002	0.100	−0.040	−0.174	0.179	0.086
	*p*-Value	**0.013**	**0.001**	0.171	0.763	0.542	0.705	0.092	0.104	0.338
Q3	*B*	−0.012	0.146	−0.002	0.003	−0.236	**0.707**	−0.034	0.098	0.164
	*p*-Value	0.053	0.367	0.802	0.777	0.295	** < 0.001**	0.812	0.531	0.181
Q4	*B*	**−0.013**	**0.347**	**0.022**	0.001	−0.126	**0.496**	0.055	0.059	0.022
	*p*-Value	**0.041**	**0.046**	**0.015**	0.947	0.600	**0.002**	0.714	0.724	0.869
Q5	*B*	**−0.013**	0.242	0.006	−0.008	−0.020	**0.285**	−0.034	−0.108	0.077
	*p*-Value	**0.009**	0.094	0.400	0.444	0.921	**0.031**	0.789	0.422	0.480
Q6	*B*	**−0.016**	**0.385**	0.004	−0.023	−0.008	**0.334**	0.024	0.211	0.245
	*p*-Value	**0.011**	**0.031**	0.676	0.064	0.972	**0.037**	0.879	0.200	0.066
Q7	*B*	−0.011	0.089	0.004	−0.025	−0.252	**0.394**	−0.248	−0.076	0.162
	*p*-Value	0.099	0.648	0.711	0.064	0.354	**0.027**	0.147	0.680	0.275
Q8	*B*	**−0.009**	0.064	-0.003	−0.002	0.009	−0.050	−0.141	0.197	0.127
	*p*-Value	**0.037**	0.590	0.664	0.840	0.956	0.646	0.175	0.078	0.158
Q9	*B*	−0.008	**0.345**	**0.013**	−0.008	−0.090	**0.269**	0.080	0.103	0.126
	*p*-Value	0.070	**0.004**	**0.041**	0.344	0.590	**0.014**	0.448	0.361	0.168
Q10	*B*	**−0.015**	0.241	0.001	−0.010	−0.223	**0.301**	0.017	0.019	**0.192**
	*p*-Value	** < 0.001**	0.050	0.907	0.251	0.193	**0.007**	0.872	0.866	**0.040**
Q11	*B*	−0.006	**0.304**	0.007	−0.006	−0.113	0.160	−0.198	0.123	**0.217**
	*p*-Value	0.187	**0.019**	0.324	0.488	0.529	0.173	0.081	0.310	**0.028**
Q12	*B*	**−0.010**	0.217	0.006	−0.009	**0.470**	**0.240**	−0.066	0.060	**0.224**
	*p*-Value	**0.025**	0.088	0.344	0.317	**0.008**	**0.038**	0.550	0.613	**0.021**

Bold values indicate signifiacant *p*-values

#### Question 9: Working

In the regression model, the weight of the question on work was associated with gender (*p* = 0.004, *B* = 0.345), height (*p* = 0.041, *B* = 0.013), and self-use of a car (*p* = 0.014, *B* = 0.269; Table 4=). A significant positive correlation between the recorded OKS score and recorded OKS weight was found for the question on working (*r* = 0.174, *p* = 0.014; Table 3).

#### Question 10: Stability

In the regression model, instability was associated with older age (*p* < 0.001, *B* = −0.015), self-use of a car (*p* = 0.007, *B* = 0.301), and previous surgery (*p* = 0.040, *B* = 0.192; Table 4). A significant positive correlation between the recorded OKS score and recorded OKS weight was found for the question on stability (*r* = 0.166, *p* = 0.018; Table 3).

#### Question 11: Shopping

Household shopping was again influenced by gender (*p* = 0.019, *B* = 0.304) and previous surgery (*p* = 0.028, *B* = 0.217; Table 4).

#### Question 12: Walking stairs

Walking down a flight of stairs was associated with older age (*p* = 0.025, *B* = −0.010), having a set of stairs at home (*p* = 0.008, *B* = 0.470), self-use of a car (*p* = 0.038, *B* = 0.248), and previous surgery (*p* = 0.021, *B* = 0.224; Table 4).

## Discussion

The most important finding of the present study is that age, driving ability, using walking aids, and working status were the most important factors for the weight of each of the questions.

There are mixed results for a possible ceiling effects of the OKS [2, 5, 10, 15, 16]. Marx et al. [10] noted that 5% of patients at 6 months and 7% of patients at 12 months post-surgery achieved the top score, indicating the presence of a ceiling and floor effect of the OKS. Hamilton et al. [17] reported 8% of top possible score in 4709 patients undergoing total knee arthroplasty (TKA). Postoperative ceiling effects for the OKS have been reported up to 27% [18] and 33% [16]. Harris et al. [2] did not find a ceiling effect for the OKS. However, in subgroup analyses males tended to score higher on postoperative OKS than females. The proportion of patients achieving postoperative top scores in males was almost double that of the female population, with 3.8% compared with 2% [2]. The results in the present study indicate differences according to gender. The questions on pain and washing were more important for female patients in the regression models. Therefore, female patients may have different expectations preoperatively and focus on different daily activities than male patients. As the OKS does not differentiate between different genders, this may result in an inherent difference in the OKS, leading to generally lower average mean scores because of the conception of questions and equal importance. A distinction between both genders and a consecutive adjustment for gender could be a possible improvement for the OKS and other PROMs.

Another ceiling effect for the OKS is described for age [6]. Harris et al. [6] found the highest ceiling percentage of 3% in the subgroup of patients between 60 and 79 years of age. These findings are also supported by the results of the presented study. Older age was the most important factor in the regression models in this study. This indicates that patients with older age are more focused on pain and night pain and focus their importance weight more on these categories. Therefore, a benefit in analyzing the pain and function subscales of the OKS separately after TKA is suggested [2]. The higher focus on pain due to older age demonstrated by the results in the presented study backs this suggestion. The equal scoring systems of the OKS might lead to ceiling effects because of missing representation of different age groups, which could be a possible target for adjustment of PROMs such as the OKS.

Ceiling and floor effects are initially addressed in the conception of a PROM [2]. In the conception of the OKS, a number of different items have been rejected [2]. However, ceiling and floor effects in PROMs are common [10]. Ceiling effects are also related to the number of items in a scale [11]. The more items a scale addresses, the less likely the patient chooses the highest or lowest response category in every single item [11]. However, the WOMAC score is outperformed by the FJS-12, which contains only half as many questions with only half as many patients achieving the best possible score [11]. In evaluating the Danish version of the FJS-12, the ceiling effect was significantly lower, with 16% compared with 37% for the OKS [15]. In scoring a PROM, the scoring systems is equally important as the quality of the question itself. The question itself can discriminate patients according to age or gender or activity level. Furthermore, an equally scaled scoring system can also lead to discrimination, as not all questions are equally important, which is demonstrated by the results presented in this study, as, e.g., the question on driving is more important for patients with self-use of a car. The FJS-12 also uses the same 5-item scoring scale for each question [11]. The questions are conceived to overcome discrimination such as in case of asking for awareness of the artificial joint while doing the patients’ favorite sports. It tries to overcome the different level of activities by asking for the patients’ favorite sport. However, in case of elderly patients, this question might impair the final score because the patients are not able to answer this question adequately, as they do not engage in sports at all.

One major advantage of the OKS lies in the simplicity in completing it [1]. Problems with possible ceiling effects or floor effects of one PROM could be addressed by using different outcome scores [3]. Some authors suggest using both joint-specific and generic health measures to assess the outcome after TKA [20, 21]. Measurements of quality of life such as the 36-Item Short Form Survey (SF-36) are commonly used in combination with PROMs [3]. However, the SF-36 was not developed specifically for total joint arthroplasty (TJA), leading to susceptibility to other influences such as pain and disability from other weight-bearing joints and other symptomatic conditions [1, 22]. Additionally, utilizing too many questionnaires can lead to response burden of the patient [23–25]. Response burden depends on the cognitive function of the patient [24] and is merely relying on the base decisions on use of instruments on the content rather than the length of the questionnaire [25].

Although pain relief and improvement in function after TJA is generally achieved to a satisfying extent for patients undergoing TJA, nuisance symptoms are generally common. As an alternative to PROMs focusing on pain or stability of the joint, newer PROMs such as the Forgotten Joint Score (FJS-12) focus on other aspects in TJA [11]. Noble et al. [19] found patients who had undergone arthroplasty to have a lower functional level than age- and sex-matched healthy controls. This indicates that the current concepts of TKA are not able to restore normal healthy joint function with an artificial joint in the near future [11, 19]. This is also an important aspect for PROMs, as it is elusive to ask questions about daily activities that are not possible or not relevant for patients after TKA. This would be an important aspect for future adjustments to new PROMs: to distinguish more clearly activities that are more important for arthroplasty patients.

Some limitations need to be noted. Firstly, the study was powered on an *r*-value of 0.3, which is low correlation. However, we found statistically significant correlation and significant predictive values for several sociodemographic aspects and several OKS questions. Secondly, the sociodemographic data of some of the categories was collected using a binary questionnaire. Even so, the outcomes even for these variables were significant in a number of instances. A further breakdown of the number of stairs at home and the number of kilometers driven would certainly provide more information. Due to a single-site data collection, the results might differ in other centers and countries.

## Conclusion

The questions in the OKS differ significantly in weight for each patient, based on sociodemographic data, such as age, self-use of a car, and employment. With these differences, the Oxford Knee Score might be limited as an outcome measure. Adjustment of the OKS that incorporates the demographic differences into the final score might be useful if the ceiling effect is to be mitigated.

## Data Availability

Data and materials are available on request.
